# Learning on the job: using Artificial Intelligence to support rapid review methods

**DOI:** 10.5195/jmla.2024.1868

**Published:** 2024-04-01

**Authors:** Kristin Rogers, Leah Hagerman, Sarah Neil-Sztramko, Maureen Dobbins

**Affiliations:** 1 readk@mcmaster.ca, Research Coordinator IV, National Collaborating Centre for Methods and Tools, School of Nursing, McMaster University, Suite 210A – 175 Longwood Rd S., Hamilton, ON, Canada, L8P 0A1; 2 hagerml@mcmaster.ca, Research Coordinator, National Collaborating Centre for Methods and Tools, McMaster University, Suite 210A – 175 Longwood Rd S., Hamilton, ON, Canada, L8P 0A1; 3 neilszts@mcmaster.ca, Assistant Professor, Health Research Methods, Evidence & Impact, McMaster University, 1280 Main St W, Hamilton, ON, Canada, L8S 4L8; 4 dobbinsm@mcmaster.ca, Professor, Scientific Director, National Collaborating Centre for Methods and Tools, School of Nursing, McMaster University, Suite 210A – 175 Longwood Rd S., Hamilton, ON, Canada, L8P 0A1

**Keywords:** Public health, rapid review, Artificial Intelligence

## BACKGROUND

The National Collaborating Centre for Methods and Tools' (NCCMT) Rapid Evidence Service [1] conducts rapid reviews on priority questions to respond to the needs of public health decision-makers. Given the vast quantity of literature available, a key challenge of conducting rapid evidence syntheses is the time and effort required to manually screen large search results sets to identify and include all studies relevant to the research question within an accelerated timeline. To overcome this challenge, the NCCMT investigated the integration of artificial intelligence (AI) technologies into the title and abstract screening stage of the rapid review process to expedite the identification of studies relevant to the research question.

The NCCMT is funded by the Public Health Agenda of Canada and affiliated with McMaster University.

## PROJECT

The NCCMT leveraged existing AI features within the DistillerSR [2] systematic review software to develop a standard approach that can be used to support relevance screening in rapid reviews on a variety of public health topics. These include DistillerSR Artificial Intelligence SYstem (DAISY) Re-Rank, Re-Rank Report, AI Screening, and Check for Screening Errors.

The DAISY Re-Rank feature has been used in 30 rapid reviews on 18 topic areas to re-order the search results set throughout title and abstract screening and prioritize references that are more likely to be relevant to the research question. This has enabled the NCCMT team to initiate the full-text screening and data extraction stages earlier in the rapid review process by quickly identifying the studies most relevant to the research question.

The Re-Rank Report feature acts as a checkpoint to inform decisions around when it is appropriate to start using the AI Screening feature by predicting the probability that all included studies have been identified. The Check for Screening Errors feature provides an additional layer of confidence that all included studies have been identified and minimizes the risk of incorrectly excluding relevant studies. The results of each of these features inform the use of the AI screening feature as a single screener or in duplicate with human manual screening.

The AI Screening feature uses a threshold-based prediction score to identify relevant or irrelevant references to the research question. The NCCMT has used the AI Screening feature on 6 rapid reviews, including three living rapid reviews. In one of the living rapid review updates, the AI Screening feature automatically excluded 80% of the search results (5,744/7,196 references). This saved a substantial amount of time during the manual title and abstract screening stage and allowed the Rapid Evidence Service team to be reallocated to full-text relevance screening, data extraction, quality appraisal, and synthesis stages earlier, thereby making faster progress to review completion.

## TECHNOLOGY

DistillerSR [2] is a literature review and evidence management software that aims to automate the literature review process.

DAISY Re-Rank uses Natural Language Processing to learn manual relevance screening patterns and apply learnings to the remaining references to predict potentially relevant studies.

Re-Rank Report predicts the total number of included studies based on previous screening patterns.

AI Screening automatically screens studies based on prediction scores.

Check for Screening Errors identifies studies that were potentially falsely excluded.

## ADVANTAGES

There are several key advantages of developing a standard process to integrate AI technologies into rapid evidence syntheses.

These include:

- Reducing staff time spent on manual title and abstract screening.
- Automatically excluding the most irrelevant references from large search results sets.
- The transferability of the process that can be used on a variety of different topic areas.
- The flexibility in how different AI technologies can be used for each individual rapid review based on confidence in the training set and accurate functioning of the AI features.

## LIMITATIONS

There are important limitations to this novel approach for using AI technologies in evidence synthesis.

These include:

- The need to retrain the AI features with each new research question, and the importance of a quality training data set.
- The reliance on human input for validation and the limited time to do multiple rounds of testing within accelerated timelines.
- A lack of consistency around appropriate thresholds for decision making, which can be moderated by applying learnings on the strengths and limitations of each of the AI features gained over many reviews.
- The inability to apply AI technologies to other stages in the rapid review process, such as full-text screening, data extraction, quality assessment, and synthesis.

## CONCLUSION

As of August 2022, NCCMT has successfully integrated a standard process using DistillerSR's AI features into rapid reviews on various public health relevant research questions to reduce the manual screening burden, saving time and resources. This has allowed for more timely access to high-quality synthesis evidence to inform public health decisions.
